# Evaluating a Global Assessment Measure Created by Standardized Patients for the Multiple Mini Interview in Medical School Admissions: Mixed Methods Study

**DOI:** 10.2196/38209

**Published:** 2022-08-30

**Authors:** Ann Blair Kennedy, Cindy Nessim Youssef Riyad, Ryan Ellis, Perry R Fleming, Mallorie Gainey, Kara Templeton, Anna Nourse, Virginia Hardaway, April Brown, Pam Evans, Nabil Natafgi

**Affiliations:** 1 Biomedical Sciences Department School of Medicine Greenville University of South Carolina Greenville, SC United States; 2 Patient Engagement Studio University of South Carolina Greenville, SC United States; 3 Family Medicine Department Prisma Health Greenville, SC United States; 4 School of Medicine Greenville University of South Carolina Greenville, SC United States; 5 Hospital Based Accreditation Accreditation Council of Graduate Medical Education Chicago, IL United States; 6 School of Medicine Columbia University of South Carolina Columbia, SC United States; 7 Prisma Health-Upstate Simulation Center School of Medicine Greenville University of South Carolina Greenville, SC United States; 8 Admissions and Registration School of Medicine Greenville University of South Carolina Greenville, SC United States; 9 Health Services, Policy, Management Department Arnold School of Public Health University of South Carolina Columbia, SC United States

**Keywords:** co-design, participatory design, medical schools, exploratory sequential mixed methods design, school admission criteria, medical students, communication, multiple mini interviews, interview, patient, student, medical school, acceptance, study design

## Abstract

**Background:**

Standardized patients (SPs) are essential stakeholders in the multiple mini interviews (MMIs) that are increasingly used to assess medical school applicants’ interpersonal skills. However, there is little evidence for their inclusion in the development of instruments.

**Objective:**

This study aimed to describe the process and evaluate the impact of having SPs co-design and cocreate a global measurement question that assesses medical school applicants’ readiness for medical school and acceptance status.

**Methods:**

This study used an exploratory, sequential, and mixed methods study design. First, we evaluated the initial MMI program and determined the next quality improvement steps. Second, we held a collaborative workshop with SPs to codevelop the assessment question and response options. Third, we evaluated the created question and the additional MMI rubric items through statistical tests based on 1084 applicants’ data from 3 cohorts of applicants starting in the 2018-2019 academic year. The internal reliability of the MMI was measured using a Cronbach α test, and its prediction of admission status was tested using a forward stepwise binary logistic regression.

**Results:**

Program evaluation indicated the need for an additional quantitative question to assess applicant readiness for medical school. In total, 3 simulation specialists, 2 researchers, and 21 SPs participated in a workshop leading to a final global assessment question and responses*.* The Cronbach α’s were >0.8 overall and in each cohort year. The final stepwise logistic model for all cohorts combined was statistically significant (*P*<.001), explained 9.2% (*R*^2^) of the variance in acceptance status, and correctly classified 65.5% (637/972) of cases. The final model consisted of 3 variables: empathy, rank of readiness, and opening the encounter.

**Conclusions:**

The collaborative nature of this project between stakeholders, including nonacademics and researchers, was vital for the success of this project. The SP-created question had a significant impact on the final model predicting acceptance to medical school. This finding indicates that SPs bring a critical perspective that can improve the process of evaluating medical school applicants.

## Introduction

People trained to act out a role consistently and repeatedly in a realistic way for active learning and assessment purposes in medical education are called standardized patients (SPs) or simulated participants [1,2]. They will not only act in the simulations but also assist in the assessment of learners. Ample evidence suggests that SPs can effectively assess and evaluate medical students, nursing students, medical residents, and other clinical learners [3-5]. They are essential stakeholders and collaborators within the medical education process who help learners at all levels meet educational objectives [1,2,6].

At our institution, our team of SPs partners with the simulation center staff to create a safe environment for learners to fail and make mistakes. The practice of medicine is complex and nuanced, which requires physicians to have the skills and abilities to work cross-culturally with patients and families to make life and death decisions every day. It is difficult to learn these needed skills simply in a classroom; practical and practiced application of the assessable skills within a simulated environment is needed. The simulated environment must not only be a place to learn and practice the skills and abilities but also to fail and make mistakes. This safe and simulated environment is the fundamental link to the education of compassionate and competent future physicians.

Simulation center demonstrations and recruitment events, as well as referrals by current SPs or staff members, allow for the incorporation of new SPs at our institution. People who become part of the simulation center team as SPs come from all walks of life and represent various demographics, work histories, and experiences. All SPs are trained to recreate the history, personality, physical findings, emotional structure, and response pattern of an actual patient accurately and consistently during a simulated experience or scenario. They are also trained to assess learner performance and provide individualized feedback to learners in a constructive manner. After training, the SPs partner with staff in delivering the medical scenarios to learners where the objectives focus on skill development. Equally important are nonmedical scenarios that focus on the development of verbal and nonverbal communication skills. In the nonmedical scenarios, they teach students through practical application, assessment, and active learning. Thereby, SPs along with the rest of the medical school faculty and staff coeducate and codevelop these learners into future physicians. Additionally, they not only help learners during their educational experiences but are also core team members during the medical school admissions process [3,7].

Many medical schools have moved toward a holistic admissions process. This process can include the assessment of applicants’ noncognitive skills such as moral reasoning and interpersonal communication through the inclusion of multiple mini interviews (MMIs) [7-17]. MMIs are designed to evaluate the applicants’ listening abilities, professionalism, ethics, empathy, integrity, cultural sensitivity/humility, problem-solving skills, and communication skills [9,10,15,18,19]. During the MMI, each applicant rotates through a series of stations where SPs evaluate their noncognitive traits [7,8,12,20-22]. SPs have been identified as a valid and reliable resource to evaluate applicants in an MMI process [3,23].

The inclusion of stakeholders in the design and implementation of interventions that impact their own population can provide more robust outcomes [24-29]. Additionally, meaningfully involving patients and the public in the co-design of interventions produces studies that are more patient-centered, less disruptive for study participants, and more accepted and valued by the study population [30-36]. The literature has shown that when SPs codevelop simulation scenarios and educational experiences, they can enhance outcomes for learners and educators [37-39]. However, we found no evidence for the inclusion of SPs in the process and development of an evaluation instrument, tool, or rubric for use during simulated interactions with learners or medical school applicants. Therefore, the purpose of this study was to describe the process and evaluate the impact of having SPs cocreate a global measurement question that assesses medical school applicants’ readiness for medical school.

## Methods

### Study Design

**Figure 1:**
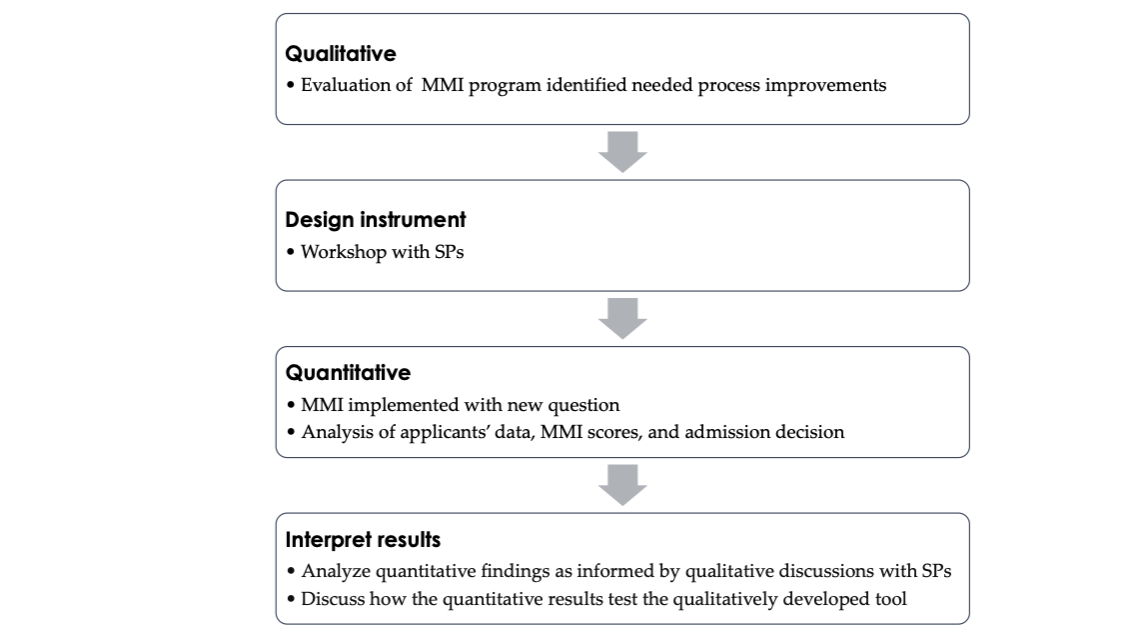
Illustration of participatory, exploratory, sequential, and mixed methods design flow. MMI: multiple mini interview; SP: standardized patient.

This study followed a participatory, exploratory, sequential, and mixed methods design (Figure 1) [40-43]. Exploratory designs are commonly used for developing new instruments or measures [43-45] and are particularly useful when a culturally responsive instrument needs to be created [43].

### The MMI Program

**Figure 2:**
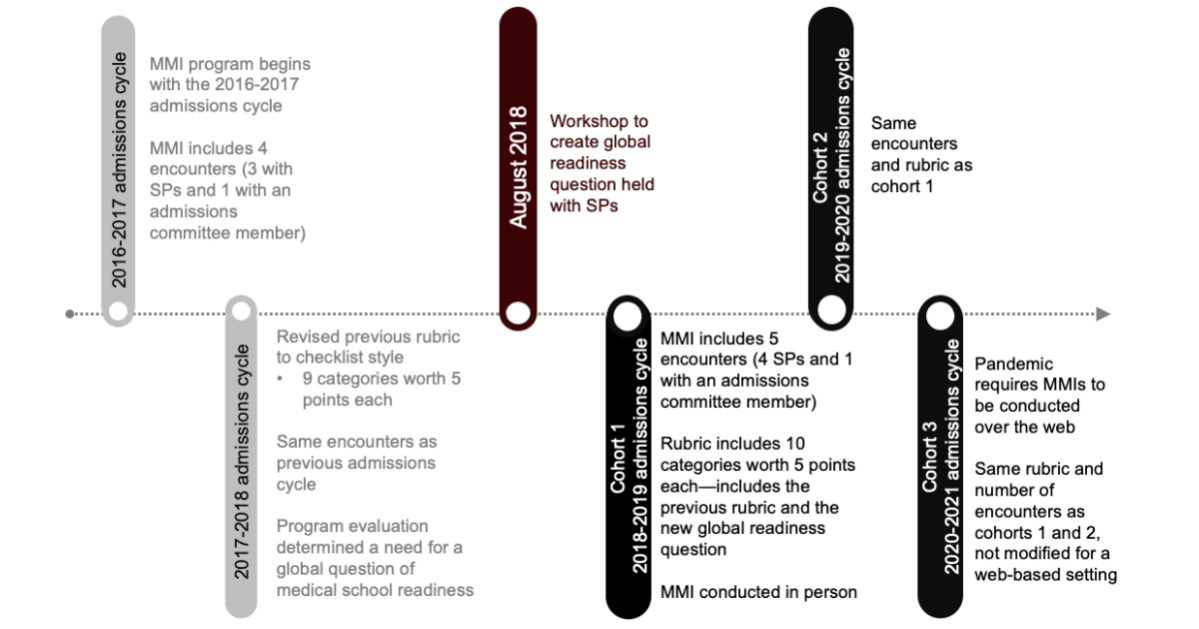
Timeline of the MMI program and evaluation beginning in 2016 through program evaluation and 3 cohorts for analysis. MMI: multiple mini interview; SP: standardized patient.

The original MMI process was implemented in the 2016-2017 admissions cycle [15], and a timeline of the program procedures and revisions can be found in Figure 2. The MMI encounters that are a part of our admissions process are delivered and evaluated by our simulations center team which includes simulations specialists and SPs. These encounters require no medical knowledge by the applicant and can simulate communication with a patient, peer, or coworker. For each MMI encounter, the applicant interacts with a single SP while being observed through a 2-way window by another independent SP in real time. At the conclusion of the encounter, the observing SP and performing SP collaboratively discuss the applicant’s performance and skills and complete the rubric to provide a quantitative outcome. The evaluation by 2 SPs helps eliminate potential bias, ensures accuracy in scoring, and streamlines the fast pace of MMIs. Furthermore, a standardized rubric and 2 SPs ensure accuracy and timely completion in the limited time allotted between encounters.

### MMI Program Evaluation

SPs, simulation center staff, and members of the admissions committee participated in an evaluation of the MMI program after the 2017-2018 cycle. This evaluation included a review of the MMI rubric and admissions committee documents for quality improvement purposes. According to evaluation results, partnering with SPs to cocreate an addition to the evaluation rubric could improve the MMI program.

### SP Workshop

For the SPs involved in the MMI during the medical school admissions process, the simulation specialists and researchers hosted a workshop in August 2018 to allow for the cocreation and improvement of the rubric. The workshop included a short didactic session and active participation by the SPs. During the didactic presentation (Multimedia Appendix 1), the researchers gave a short presentation to (1) review the rubric used in the previous year, (2) present reasons for creating the new question, (3) explain the basic survey design [46], and (4) discuss the importance of stakeholder engagement in this type of work.

Upon completing the presentation, the SPs were divided into small groups and given approximately 30 minutes to create 1 question and the corresponding responses. The created questions were written and displayed on large pieces of paper placed on the walls and on 2 screens in the room. Subsequently, everyone was reassembled into a large group to discuss the developed questions. Voting and further discussion led to a single SP-created question. Once the question had been finalized, the SPs were then divided into 2 groups to write response options for the newly created question. Collaborative discussion, voting, and editing led to a co-designed set of response options.

### Quantitative Data Collection and Analysis

The quantitative data were collected from various sources, including 1084 medical school applications, MMI process forms, and admission status for 3 cohorts: (1) 2018-2019, (2) 2019-2020, and (3) 2020-2021. Applicants’ demographics were gathered from school applications. Averages of the 4 SP encounters for each MMI category were provided for individual applicants. Admission status was categorized into 3 categories: accepted, declined, or wait-listed (reviewed applicants awaiting consideration from those who withdrew their application prior to acceptance). The wait-listed applicants were excluded from the regression models. In all, 3 applicants did not have admissions or matriculations status listed and were excluded from analysis.

### Statistical Analysis

Quantitative data analyses were performed using SPSS software (version 26; IBM Corp). Descriptive statistics included frequencies, means and SDs, and percentages. The internal consistency reliability of the MMI was measured using a Cronbach α test evaluating all 10 variables within the MMI: (1) opening the encounter, (2) empathy, (3) nonverbal behavior, (4) verbal behavior, (5) listens well, (6) therapeutic relationship, (7) negotiation of the plan, (8) closing the encounter, (9) rank of readiness, and (10) admissions committee member. To determine if the SPs may be influenced by the demographics of the applicants, the applicants’ age, sex, and underrepresented in medicine (URM) status (dichotomous yes/no) were regressed (multiple linear regression) on the average MMI score (excluding the admissions committee member score). Finally, we sought to determine if medical school admissions status could be predicted based upon the variables within the SP-scored MMI (9 variables). A forward stepwise binary logistic regression was used to identify possible predictors of acceptance status (accepted or declined) out of the following MMI candidate variables: opening the encounter, empathy, nonverbal behavior, verbal behavior, listens well, therapeutic relationship, negotiation of the plan, closing the encounter, and rank of readiness. The forward logistic regression used the likelihood-ratio test to enter or remove variables from the model.

### Ethics Approval

Ethical oversight of the project was conducted by the Institutional Review Board at the University of South Carolina (Pro00069266).

## Results

### MMI Program Evaluation

Feedback from the SPs, simulation center staff, and admissions department revealed that the checklist rubric (Multimedia Appendix 2) was easy for SPs to use and for the admissions committee to integrate into their decision-making process. In contrast, some SPs would write extensive qualitative comments about applicants, whereas others would leave few or no notes on those they interviewed. Additionally, it was revealed that the admissions committee was not using these written comments by the SPs in their applicant deliberations. Therefore, adding a single MMI rubric item that evaluates applicants based on their interactions with SPs during the scenario might reduce the SPs’ need to write qualitative comments and provide additional information for the admissions committee. To this end, a workshop was planned and implemented to develop a question and responses to provide a global assessment of a medical school applicant.

### SP Workshop and Global Measure Development

In attendance at the workshop were 3 simulation specialists, 2 researchers, and 21 SPs. The mean age of the SPs was 53.1 (SD 12.43; range 27-70) years, and a majority were female (16/21, 76%) and had a bachelor’s degree or higher (18/21, 86%). The 6 SP-created questions are listed in Table 1. The SPs identified that 3 of the 6 questions were dichotomous in nature and concluded that if selected, these 3 questions would not be a useful global measure assessment tool. Additionally, they identified certain words that they deemed to be either useful or problematic, such as “successful,” “characteristics,” “1st year” (vs medical student in general), “ability,” and “readiness.” Several SPs deliberated over the ways to strengthen the questions. For example, the SPs suggested adding “Based on this interaction,” to the beginning of the second question to help the person answering the question focus on the interaction with the applicant and not the applicant’s qualities overall. Additionally, the question of whether communication skills could be developed or if they are innate and unchangeable was discussed.

At the end of the discussion, voting resulted in questions 6 and 4 receiving 10 and 9 votes, respectively (2 of the 21 SPs had to leave the workshop before the voting process). Additional discussion led to a “tie-breaking vote,” yielding question 6 as the top choice. The SPs, however, determined that both questions had some aspects they wanted to emphasize. Thus, a single question was merged from both, which resulted in the combined global question: *“Based on the candidate’s communication and interpersonal skills, rate this candidate’s readiness for medical school.”*

As shown in Table 2, both groups developed response options for the global readiness question. There was a brief discussion before a vote was taken, and rubric 2 was chosen with some minor group edits. The bottom 2 scale items were modified to add *readiness* in light of the question. The word *adequate* was replaced by the word *proficient* in the third scale item. On the *advanced* scale item, the words following the slash may be listed interchangeably instead of in the specific order listed.

**Table 1:** Original questions created by standardized patients.

Group number	Question
1	Based on this interaction, how successful do you think this applicant is likely to be as a medical student?
2	Will this candidate be a successful 1st year medical student?
3	Do you think this student demonstrates the characteristics of a successful medical student?
4	How well did this candidate demonstrate communication and interpersonal skills which will allow him/her to succeed in medical school?
5	Do you think the applicant will be a successful medical student?
6	Rate this candidate’s readiness for medical school.

**Table 2:** Standardized patient–developed response options for the developed readiness question.

Ranking score	Response options set 1	Response options set 2	Final response options set
Rank of 5	Exemplary	Exceptional/extraordinary readiness	Exceptional/extraordinary readiness
Rank of 4	Ready with minor concerns	Advanced/strong and engaged	Advanced/strong and engaged
Rank of 3	Ready with some concerns	Adequate/addresses basics	Proficient/addresses basics
Rank of 2	Serious concerns	Minimal	Minimal readiness
Rank of 1	Not ready	Did not demonstrate	Did not demonstrate readiness

### Quantitative Results

#### Medical School Applicants Characteristics

Table 3 summarizes the characteristics of the 1084 medical school applicants who were selected and invited for an interview by the 3 corresponding cohort years and as a whole. Overall, a little over half (589/1084, 54.3%) of the interviewees were female, and their average age was 24.7 (SD 2.87) years. Of the 1084 interviewees, 20.1% (n=218) were from racial/ethnic communities considered as URM, and 12.5% (n=135) identified as African American or Black. In addition to the interviewees’ characteristics, Table 3 presents the average score and SD of each of the MMI categories for each of the 3 cohorts. The average MMI score for rank of readiness (ie, the SP-created question: “Based on the candidate’s communication and interpersonal skills, rate this candidate’s readiness for medical school”) increased over time from 3.38 (SD 0.53) to 3.52 (SD 0.48) out of 5, with 5 being exceptional or extraordinary readiness. In contrast, the MMI score of the admissions committee member (ie, painting/image discussion) decreased over time from 3.64 (SD 0.92) to 3.45 (SD 0.82). Of those interviewed, 52% (199/383), 51.9% (187/360), and 60.7% (207/341) were offered admissions to the medical school in cohorts 2018-2019, 2019-2020, and 2020-2021, respectively.

**Table 3:** Descriptive statistics of interviewees by cohort year.

Variable			2018-2019 (n=383)		2019-2020 (n=360)		2020-2021 (n=341)		Total (N=1084)
**Sex, n (%)**									
	Female	201 (52.5)		192 (53.3)		196 (57.5)		589 (54.3)	
	Male	182 (47.5)		166 (46.1)		145 (42.5)		493 (45.5)	
	Prefer not to answer	0 (0)		—^a^		0 (0)		—	
Age (years), mean (SD)			25.44 (2.95)		24.92 (2.62)		23.68 (2.74)		24.71 (2.87)
**Underrepresented in medicine, n (%)**									
	Total	57 (14.9)		73 (20.3)		88 (25.8)		218 (20.1)	
	African American or Black	32 (8.4)		43 (11.9)		60 (17.6)		135 (12.5)	
	American Indian or Alaska Native	—		—		—		8 (0.7)	
	Hispanic or Latinx	22 (5.7)		27 (7.5)		23 (6.7)		72 (6.6)	
	Multiracial or mixed race	—		—		—		—	
Opening the encounter, mean (SD)			4.13 (0.60)		4.28 (0.61)		4.18 (0.56)		4.19 (0.59)
Empathy, mean (SD)			3.85 (0.75)		3.93 (0.76)		4.12 (0.61)		3.96 (0.72)
Nonverbal behavior, mean (SD)			4.57 (0.44)		4.49 (0.56)		4.24 (0.63)		4.44 (0.56)
Verbal behavior, mean (SD)			3.97 (0.44)		3.93 (0.62)		4.10 (0.50)		4.00 (0.57)
Listens well, mean (SD)			3.69 (0.51)		3.71 (0.51)		4.02 (0.50)		3.80 (0.53)
Therapeutic relationship, mean (SD)			4.30 (0.69)		4.24 (0.73)		4.35 (0.61)		4.30 (0.68)
Negotiation of the plan, mean (SD)			2.03 (0.64)		2.14 (0.62)		2.75 (0.68)		2.30 (0.68)
Closing the encounter, mean (SD)			3.46 (0.63)		3.54 (0.66)		3.71 (0.57)		3.56 (0.63)
Rank of readiness, mean (SD)			3.38 (0.53)		3.46 (0.57)		3.52 (0.48)		3.45 (0.53)
Admissions committee member score (painting/image discussion), mean (SD)			3.64 (0.92)		3.36 (0.92)		3.45 (0.82)		3.48 (0.90)
**Acceptance status, n (%)**									
	Accepted	199 (52)		187 (51.9)		207 (60.7)		593 (54.7)	
	Declined	122 (31.9)		146 (40.6)		111 (32.6)		379 (35)	
	Wait-listed	62 (16.2)		27 (7.5)		23 (6.7)		112 (10.3)	

^a^Cells with n≤5 were suppressed to protect the identity of the individuals.

#### Evaluation of MMI

The 10-variable MMI had a high level of internal consistency overall and in each cohort year as determined by Cronbach α’s of 0.877 (all years combined), 0.89 (2018-2019), 0.90 (2019-2020), and 0.87 (2020-2021).

#### Regression Models for MMI Scores

We ran a multiple regression to predict the average MMI score from age, sex, and URM status. The multiple regression model for all cohorts predicted the average MMI score (*P*<.001, *R*^2^=.035). Of the 3 variables, 2 (sex and URM status) added statistical significance to the prediction (*P*<.001 and *P*=.003, respectively). The results of the multiple regression models of the individual cohorts can be found in Table 4.

Although MMI average scores are used for admission committee discussions, this analysis is aimed at determining how much impact each of the variables within the MMI may have on the acceptance decision—specifically, to determine if the readiness for medical school should be selected as a predictor variable. A forward stepwise binomial logistic regression was performed to ascertain the effects of the MMI variables (opening the encounter, empathy, nonverbal behavior, verbal behavior, listens well, therapeutic relationship, negotiation of the plan, closing the encounter, and rank of readiness) on the likelihood that applicants would be accepted to medical school. Models were built for all cohorts combined and each cohort individually. Table 5 contains results from all groups and models.

**Table 4:** Multiple regression results for MMI average.

MMI^a^ average			Unstandardized coefficient							Standardized coefficient (β)			*R* ^2^				Δ*R*^2^				*P* value	
			B		95% CI		SE															
**2018-2019 cohort model**														0.08				0.07				<.001
	Constant	2.63		2.19-3.06		0.22		N/A^b^											<.001			
	Sex^c^	0.16		0.07-0.25		0.05		0.17											<.001			
	Age	0.03		0.02-0.05		0.01		0.22											<.001			
	URM^d^ status	0.08		–0.05 to 0.21		0.07		0.06											.21			
**2019-2020 cohort model**														0.04				0.04				<.001
	Constant	3.59		3.07-4.10		0.26		N/A											<.001			
	Sex^c^	0.17		0.07-0.27		0.05		0.18											<.001			
	Age	-0.00		–0.02 to 0.02		0.01		-0.02											.72			
	URM status	0.12		0.01-0.26		0.06		0.11											.04			
**2020-2021 cohort model**														0.017				0.009				.12
	Constant	3.65		3.18-4.12		0.24		N/A											<.001			
	Sex^c^	0.11		0.01-0.21		0.05		0.11											.04			
	Age	0.00		–0.02 to 0.02		0.01		0.02											.76			
	URM status	0.06		–0.06 to 0.17		0.06		0.06											.32			
**Total model**														.035				.032				<.001
	Constant	3.38		3.11-3.64		0.14		N/A											<.001			
	Sex^c^	0.15		0.09-0.20		0.03		0.15											<.001			
	Age	0.01		–0.002 to 0.02		0.01		0.05											.14			
	URM status	0.11		0.04-0.18		0.04		0.09											.003			

^a^MMI: multiple mini interview.

^b^N/A: not applicable.

^c^The 2 “prefer not to answer” responses for sex were removed from analysis.

^d^URM: underrepresented in medicine.

**Table 5:** Forward stepwise logistic regression predicting likelihood of acceptance to medical school.

Group, model				B		SE		Exp(B)		95% CI for Exp(B)
**2018-2019 cohort**										
	**Step 1**									
		Rank of readiness	1.083		.246		2.954		1.825-4.782	
		Constant	–3.162		.830		.042		N/A^a^	
**2019-2020 cohort**										
	**Step 1**									
		Rank of readiness	.948		.212		2.580		1.702-3.912	
		Constant	–3.023		.739		.049		N/A	
	**Step 2**									
		Negotiating the plan	.547		.215		1.728		1.133-2.637	
		Rank of readiness	.685		.234		1.983		1.253-3.139	
		Constant	–3.275		.752		.038		N/A	
**2020-2021 cohort**										
	**Step 1**									
		Therapeutic relationship	.787		.201		2.196		1.480-3.258	
		Constant	–2.791		.878		.061		N/A	
	**Step 2**									
		Opening the encounter	–.583		.272		.558		.328-.951	
		Therapeutic relationship	1.093		.251		2.984		1.823-4.884	
		Constant	–1.685		1.020		.185		N/A	
	**Step 3**									
		Opening the encounter	–.792		.296		.453		.254-.808	
		Empathy	.552		.278		1.736		1.006-2.997	
		Therapeutic relationship	.834		.282		2.303		1.325-4.000	
		Constant	–1.940		1.035		.144		N/A	
**All cohorts combined**										
	**Step 1**									
		Empathy	.688		.097		1.989		1.644-2.406	
		Constant	–2.265		.388		.104		N/A	
	**Step 2**									
		Empathy	.414		.132		1.513		1.169-1.958	
		Rank of readiness	.552		.183		1.737		1.213-2.489	
		Constant	–3.087		.479		.046		N/A	
	**Step 3**									
		Opening the encounter	–.334		.155		.716		.529-.971	
		Empathy	.519		.141		1.681		1.275-2.216	
		Rank of readiness	.690		.195		1.993		1.360-2.921	
		Constant	–2.573		.533		.076		N/A	

^a^N/A: not applicable.

For the 2018-2019 cohort, the model was statistically significant, (*χ*^2^_1_=21.33; *P*<.001). The model explained 8.7% (Nagelkerke *R*^2^) of the variance in acceptance status and correctly classified 64.2% (206/321) of cases. Sensitivity was 89.9%, specificity was 22.1%, positive predictive value was 65.3%, and negative predictive value was 57.4%. Rank of readiness was the only variable in the model.

For the 2019-2020 cohort, the final model was statistically significant (*χ*^2^_2_=28.59; *P*<.001). The model explained 11% (Nagelkerke *R*^2^) of the variance in acceptance status and correctly classified 64.3% (214/333) of cases. Sensitivity was 77.5%, specificity was 47.3%, positive predictive value was 65.3%, and negative predictive value was 62.2%. The final model (model 2) consisted of 2 statistically significant MMI variables: rank of readiness and negotiating the plan. Rank of readiness was associated with the greatest increase in the likelihood of being accepted to medical school.

For the 2020-2021 cohort, the final model was statistically significant (*χ*^2^_3_=24.97; *P*<.001). The model explained 10.4% (Nagelkerke *R*^2^) of the variance in acceptance status and correctly classified 68.2% (217/318) of cases. Sensitivity was 91.3%, specificity was 25.2%, positive predictive value was 69.4%, and negative predictive value was 60.9%. The final model (model 3) consisted of 3 statistically significant MMI variables: opening the encounter, empathy, and therapeutic relationship. Therapeutic relationship was associated with the greatest increase in the likelihood of being accepted to medical school.

The final model for all cohorts combined was statistically significant (*χ*^2^_3_=67.95; *P*<.001). The model explained 9.2% (Nagelkerke *R*^2^) of the variance in acceptance status and correctly classified 65.5% (637/972) of cases. Sensitivity was 87.9%, specificity was 30.6%, positive predictive value was 66.5%, and negative predictive value was 61.7%. The final model (model 3) consisted of 3 statistically significant MMI variables: empathy, rank of readiness, and opening the encounter. Rank of readiness was added in model 2 and was associated with the greatest increase in the likelihood of being accepted to medical school in the final model.

## Discussion

### Principal Findings

In this study, we demonstrated that SPs were able to develop a useful, credible, and relevant measure that can help the medical school admissions committee evaluate applicants beyond academic achievements. Although the MMI is only 1 portion of data used in a holistic review of medical school applicants, it can be useful to examine the impact and process of the MMI in the medical school admissions. Additionally, the created global readiness question that was incorporated into the MMI process contributed to selecting candidates for medical school.

Beginning with the 2018-2019 application cycle, simulation specialists integrated the revised rubric with the readiness question into training and conducting the MMI. This process was successfully adopted by the SPs involved in the MMI. As the SPs began using the new method of quantitatively scoring the applicant’s readiness, they found this 1 question to be more efficient than providing qualitative feedback for each applicant. Since there are only about 3 minutes between applicant encounters, the readiness question allows SPs to provide more concise feedback without being rushed.

Including stakeholders in the development of surveys and assessment instruments has been useful in other settings outside of simulation centers and has led to credible and relevant tools [47-50]. In this setting, working with SPs to create the rank of readiness question revealed what could be appropriately assessed through the lens of the SPs. The creation of the question through patient and public involvement yielded a more objective and standardized measure for scoring purposes. Additionally, stakeholder involvement contributed to a sense of value and co-ownership in the admissions process. Furthermore, by codeveloping this rubric item with the SPs, we built on their previous MMI skills, allowed item development to be iterative, allowed SPs to own the process, and accommodated their scoring needs. Incorporating these and other processes have been found to be associated with higher rates of positive research outcomes in cocreated projects [28,29].

The results of the workshop positively impact the simulation specialists providing a standardized scoring opportunity for all applicants. Training for standardization is critical in this role. Due to the collaborative nature of the workshop, the simulation specialists were able to emphasize the value of the SPs’ voices and consistent use of the evaluation tool, which is consistent with findings in other settings [27,51]. Moreover, the involvement of the SPs in the development of the question facilitates coaching of new SPs by simulation specialists to use this question to validate or summarize the data from the other rubric sections.

Our results showed that the MMI overall and the readiness question specifically were able to predict medical school acceptance. This finding is consistent with previous findings showing that SPs are able to assess students’ communication skills [52-55]. Furthermore, our findings are consistent with previous results showing that the SPs were unbiased in their assessment of the interviewees [10,56]. The observed interviewees’ demographics (age, sex, and race) predicted only 3% of the SPs’ MMI overall score. Although the findings were statistically significant, the predicted amount of change within the different demographic variables was relatively small. There is a possibility that bias was reduced because 2 SPs scored each interviewee collaboratively.

Although the MMI rank of readiness score was statistically significant for predicting an applicant’s acceptance to medical school overall and for the 2018-2019 and 2019-2020 cohorts, this was not the case for the 2020-2021 cohort. One potential explanation for this lack of significance is that the entire cycle was conducted in a web-based format due to the COVID-19 pandemic. The rubric was not changed to reflect the web-based interview environment, which could potentially account for some of the differences. For example, students were automatically given points for elements that could not be performed in a web-based environment (eg, knocking on door prior to entering). Additionally, nonverbal behavior was challenging to assess, as illustrated by score differences between cohorts with in-person or web-based MMIs (Multimedia Appendix 2). Although medical schools have faced many challenges in moving to a web-based interview format, it has been found that web-based interviews can be as reliable as traditional interviews in making sound decisions on applicants [57]. More research is needed to assess the impacts of the COVID-19 pandemic on medical school admissions and web-based SPs encounters.

### Limitations

Despite the SPs’ ability to create a measure, several other factors play a role in the admissions selection process beyond the MMI including, but not limited to, grade point average, Medical College Admission Test scores, recommendation letters, personal statements, and distance traveled (overcoming adversity or obstacles). These additional factors were not included in the models. Although the SP portion of the MMI excelled at predicting acceptance, specificity was low overall and for each cohort. Other elements of the application may have a greater impact on the applicants who were declined. Although the actual results of the statistical analysis might not be generalizable to other settings, lessons learned from the process for having patient and public involvement when creating a measurement instrument can benefit other institutions.

### Future Implications

The standard rubric used for MMI may need to be revised to remove items that cannot be performed over the web (eg, knocking on door, appropriate touch, and sustaining personal space) for admission years when web-based MMI is the standard. This potential, revised rubric could also be used to assess learners during telehealth encounters, and the new rubric would need to be evaluated. Additionally, the MMI rubric without the final readiness question is currently being used as the communication rubric for assessing learners during standard SP encounters and Objective Structured Clinical Examinations. The global question is specifically intended for SPs to determine applicant readiness for medical school, so it should not be used for students who have matriculated. However, it could be beneficial to replicate the workshop with SPs to identify a global question for the communication rubric. Finally, an additional investigation could be conducted to see how matriculating students’ MMI scores compare with the future communication scores throughout their time in medical school.

### Conclusion

The collaborative nature of this project between stakeholders, including nonacademics and researchers, was vital for the success of this project. This study shows that SPs bring a critical perspective that can improve the admissions process of evaluating medical school applicants through the MMI process. They also can further incorporate themselves as team members by cocreating an effective global question to improve the evaluation of the applicants.

## Appendix

Multimedia Appendix 1 Workshop presentation for standardized patients to create a global assessment scale for the multiple mini interview.

Multimedia Appendix 2 Multiple mini interview rubric, preworkshop and postworkshop.

## Abbreviations

MMI: multiple mini interview
SP: standardized patient
URM: underrepresented in medicine
