# Supplementary material for: Evaluating a Global Assessment Measure Created by Standardized Patients for the Multiple Mini Interview in Medical School Admissions: Mixed Methods Study
Source: J Particip Med. 2022 Aug 30;14(1):e38209. doi: 10.2196/38209 (PMC9472042; doi:10.2196/38209)
Supplement: Multimedia Appendix 1 [file jopm_v14i1e38209_app1.pdf]

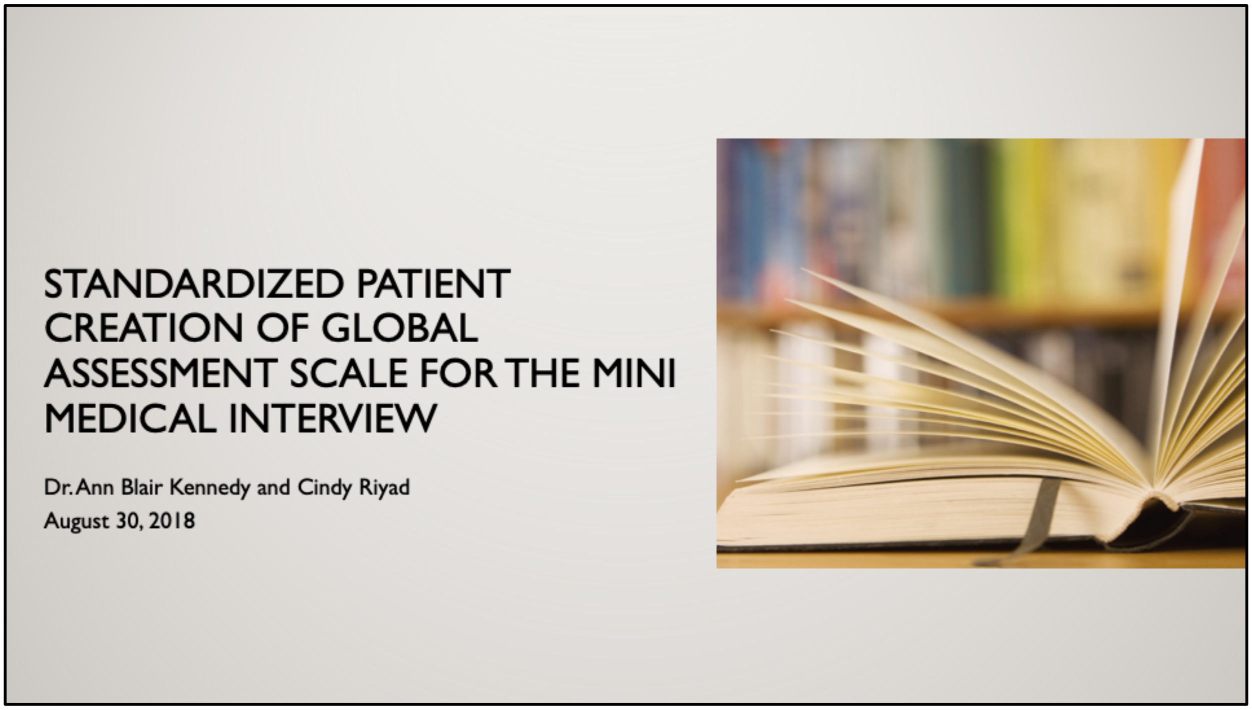

NOTE:
To change the image on this slide, select the picture and delete it. Then click the Pictures icon in the placeholder to insert your own image.

PURPOSE

CREATE A QUESTION AND RESPONSES TO PROVIDE A GLOBAL ASSESSMENT OF A MEDICAL SCHOOL APPLICANT

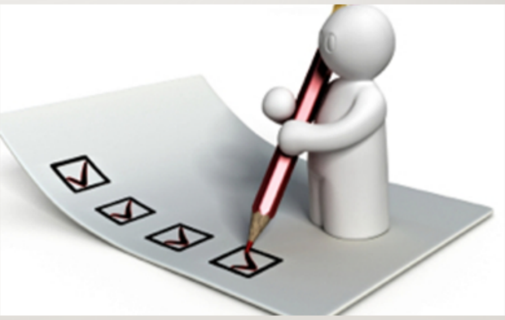

MMI CHECKLIST FOR SP TO EVALUATE APPLICANT

**Encounter Opening:**	**Empathy:**	**Non-verbal behavior:**
❑Knocked on door prior to entering<br>❑Greeted SP appropriately based on scenario<br>❑Displayed comfortable level of eye contact<br>❑Started conversation well<br>❑Exuded confidence	❑Expressed genuine concern<br>❑Offered encouraging/supportive statements<br>❑Withheld judgment<br>❑Fostered respect<br>❑Connected with/Inspired SP	❑Demonstrated appropriate facial expressions based on scenario<br>❑Exhibited relaxed, open body language<br>❑Maintained comfortable level of eye contact throughout encounter<br>❑Sustained comfortable personal space<br>❑Touched SP appropriately

MMI CHECKLIST FOR SP TO EVALUATE APPLICANT

Verbal Behavior:	Listens well:	Therapeutic relationship:
❑Asked open-ended questions<br>❑Allowed SP to tell their story<br>❑Communicated using appropriate language<br>❑Refrained from confrontation (non-judgmental)<br>❑Asked for SPs input (shared-decision making)	❑Abstained from interrupting<br>❑Offered undivided attention<br>❑Demonstrated understanding (verbal encourager)<br>❑Reflected back (paraphrased/restated both the feelings/words of the SP)<br>❑Listened to understand and not to respond	❑Demonstrated sincerity<br>❑Adjusted non-verbal behavior to match SP demeanor<br>❑Impressed upon SP a feeling of physical security (I feel safe with this individual)<br>❑Impressed upon SP a feeling of emotional security (I trust this individual)<br>❑Offered unconditional acceptance of the SP

MMI CHECKLIST FOR SP TO EVALUATE APPLICANT

**Negotiation of Plan:**	**Encounter Closing:**
❑Solicited SP input to plan<br>❑Negotiated plan/next steps<br>❑Discussed and/or Summarized plan/next steps<br>❑Requested SP to provide understanding of plan (Teach-Back)<br>❑Clarified/Corrected understanding	❑Paced encounter well<br>❑Expressed appreciation of SP story<br>❑Offered non-verbal gestures to close encounter<br>❑Questioned SP if there were additional concerns/needs<br>❑Closed encounter appropriately

“WHEN WAS THE LAST TIME YOU BOUGHT MILK?”

- Do you specifically mean ME or do you mean the person who usually buys milk in my household?
- Or do you care more about the fact that someone, anyone, in my household bought milk, whether sometimes it’s me, my spouse, my kids, my parents, a relative, or a boarder.
- Wait, do you care if the milk was purchased? Or could it be that we have an arrangement whereby we don’t actually pay for milk? Perhaps people who live on a farm with dairy cows, or people who own a convenience store?
- Do you mean only cow milk? What about milk from goats, sheep, buffalo, camel, reindeer? Or what about milk-substitutes from nuts or plants like soy, almond, rice, coconut that are labeled as milk? Were you really trying to figure out if we put a liquid on cereal?
- Does chocolate milk in a carton count? Or strawberry or banana milk?
- What if I completely forgot I bought milk last week?
- Or that I completely misread that sentence as ‘brought’ not ‘bought’ and I’ve never actually bought milk?

CREATING SURVEYS

- Good survey questions will create valid and reliable measurement of the topic of interest
- The responses are just as important as the question stem

QUESTION CREATION

FROM THE SURVEY CHECKLIST (MANIFESTO) GEHLBACH AND ARTINO (2018)

- **Avoid formatting items as statements with agree–disagree response options…**
- **…and use questions with construct specific response options instead**
  - "How much did you enjoy your biochemistry class?"
    - Did not enjoy at all
    - Enjoyed a little bit
    - Enjoyed somewhat
    - Enjoyed quite a bit
    - Enjoyed a tremendous amount
- **Use positive language**

CRAFTING RESPONSES
FROM THE SURVEY CHECKLIST (MANIFESTO) GEHLBACH AND ARTINO (2018)

- **Choose an appropriate number of response options – *we want 5***
- **Label all response options**

Box 2

Illustration of the Contrast Between Item Formats That Do and Do Not Provide Fully Labeled Response Options

By providing a verbal label for each response option (as opposed to leaving some options blank), survey designers help each option seem equally viable and clarify the meaning of each option. To illustrate, the item asking: "How skilled are you at suturing?" will work better followed by these response options:

not at all skilled	slightly skilled	moderately skilled	quite skilled	extremely skilled

…as opposed to these response options:

not at all skilled				extremely skilled

YOUR TURN

- Create your question stem and your responses
