# Supplementary material for: Evaluating a Global Assessment Measure Created by Standardized Patients for the Multiple Mini Interview in Medical School Admissions: Mixed Methods Study
Source: J Particip Med. 2022 Aug 30;14(1):e38209. doi: 10.2196/38209 (PMC9472042; doi:10.2196/38209)
Supplement: Multimedia Appendix 2 [file jopm_v14i1e38209_app2.pdf]

MMI Rubric Pre-Workshop with SP

MMI Checklist for SP to Evaluate Applicant

1. **Name of Applicant**
2. **Initials of SP**
3. **MMI Case Name**

Encounter Opening:

- ☐ Knocked on door prior to entering
- ☐ Greeted SP appropriately based on scenario
- ☐ Displayed comfortable level of eye contact
- ☐ Started conversation well
- ☐ Exuded confidence

Empathy:

- ☐ Expressed genuine concern
- ☐ Offered encouraging/supportive statements
- ☐ Withheld judgment
- ☐ Fostered respect – encouraged mutual respect
- ☐ Connected with/Inspired SP

Non-verbal behavior:

- ☐ Demonstrated appropriate facial expressions based on scenario
- ☐ Exhibited relaxed, open body language
- ☐ Maintained comfortable level of eye contact throughout encounter
- ☐ Sustained comfortable personal space
- ☐ Touched SP appropriately

Verbal Behavior:

- ☐ Asked open-ended questions
- ☐ Allowed SP to tell their story
- ☐ Communicated using appropriate language
- ☐ Refrained from confrontation (non-judgmental)
- ☐ Asked for SPs input (shared-decision making)

Listens well:

- ☐ Abstained from interrupting
- ☐ Offered undivided attention
- ☐ Demonstrated understanding (verbal encourager)
- ☐ Reflected back (paraphrased/restated both the feelings/words of the SP)
- ☐ Listened to understand and not to respond

Therapeutic relationship:

- ☐ Demonstrated sincerity
- ☐ Adjusted non-verbal behavior to match SP demeanor
- ☐ Impressed upon SP a feeling of physical security (I feel safe with this individual)
- ☐ Impressed upon SP a feeling of emotional security (I trust this individual)
- ☐ Offered unconditional acceptance of the SP

Negotiation of Plan:

- ☐ Solicited SP input prior to offering plan
- ☐ Negotiated plan/next steps
- ☐ Discussed and/or Summarized plan/next steps
- ☐ Requested SP to provide understanding of plan (Teach-Back)
- ☐ Clarified/Corrected understanding

Encounter Closing:

- ☐ Paced encounter well
- ☐ Expressed appreciation of SP story
- ☐ Offered non-verbal gestures to close encounter
- ☐ Questioned SP if there were additional concerns/needs
- ☐ Closed encounter appropriately

MMI Rubric Post Workshop with SP

1. Name of Applicant*

2. Initials of Standardized Patient*

3. MMI Case Name*

4. Encounter Opening:

Knocked on the Door prior to entering

Greeted SP appropriately based on scenario

Displayed comfortable level of eye contact

Started conversation well

Exuded confidence

5. Empathy:

Expressed genuine concern

Offered encouraging/supportive statements

Withheld judgment

Fostered respect

Connected with/Inspired SP

6. Non-verbal Behavior:

Demonstrated appropriate facial expressions based on scenario

Exhibited relaxed, open body language

Maintained comfortable level of eye contact throughout encounter

Sustained comfortable personal space

Touched SP appropriately

7. Verbal Behavior:

Asked open-ended questions

Allowed SP to tell their story

Communicated using appropriate language

Refrained from confrontation (non-judgmental)

Asked for SP's input (shared decision making)

8. Listens Well:

Abstained from interrupting

Offered undivided attention

Demonstrated understanding (verbal encourager)

Reflected back (paraphrased/restated both the feelings/words of SP)

Listened to understand and not to respond

9. Therapeutic relationship:

Demonstrated sincerity

Adjusted non-verbal behavior to match SP demeanor

Impressed upon SP a feeling of physical security (I feel safe with this individual)

Impressed upon SP a feeling of emotional security (I trust this individual)

Offered unconditional acceptance of the SP

10. Negotiation of Plan:

Solicited SP input to plan

Negotiated plan/next steps

Discussed and/or summarized plan/next steps

Requested SP to provide understanding of plan (Teach-back)

Clarified/corrected understanding

11. Encounter Closing:

Paced encounter well

Expressed appreciation of SP story

Offered non-verbal gestures to close encounter

Questions SP if there were additional concerns/needs

Closed encounter appropriately

12. Based on the candidate's communication and interpersonal skills, rate this candidate's readiness for medical school. *

Rank of 1 - Did not demonstrate

Rank of 2 - Minimal readiness

Rank of 3 - Proficient/addresses basics

Rank of 4 - Advanced/strong and engaged

Rank of 5 - Exceptional/extraordinary readiness

13. Additional Comments from Standardized Patient about this Applicant
